# Prevalence and Clinicoradiopathological Characterization of H3 K27-Altered Diffuse Midline Gliomas in Adults—A Retrospective Observational Study

**DOI:** 10.3390/pathophysiology33010021

**Published:** 2026-03-14

**Authors:** Kristof Babarczy, Bence L. Radics, Lili Kiss, Alexandra Graczer, Bence Nagy, Sandor Dosa, Gyongyi Kelemen, Marton Balazsfi, Pal Barzo, Andras Voros, Peter Klivenyi, Levente Szalardy

**Affiliations:** 1 Department of Neurology, Albert Szent-Györgyi Medical School, Albert Szent-Györgyi Clinical Center, University of Szeged, Semmelweis u. 6, H-6725 Szeged, Hungary; babarczy.kristof@med.u-szeged.hu (K.B.); kisslili050@gmail.com (L.K.); klivenyi.peter@med.u-szeged.hu (P.K.); 2 Department of Pathology, Albert Szent-Györgyi Medical School, Albert Szent-Györgyi Clinical Center, University of Szeged, Állomás utca 1, H-6725 Szeged, Hungary; radics.bence@med.u-szeged.hu (B.L.R.); graczer.alexandra@med.u-szeged.hu (A.G.); nagy.bence@med.u-szeged.hu (B.N.); dosa.sandor@med.u-szeged.hu (S.D.); voros.andras@med.u-szeged.hu (A.V.); 3 Department of Oncotherapy, Albert Szent-Györgyi Medical School, Albert Szent-Györgyi Clinical Center, University of Szeged, Korányi fasor 12, H-6720 Szeged, Hungary; kelemen.gyongyi@med.u-szeged.hu; 4 Department of Neurosurgery, Albert Szent-Györgyi Medical School, Albert Szent-Györgyi Clinical Center, University of Szeged, Semmelweis u. 6, H-6725 Szeged, Hungary; marton.balazsfi@med.u-szeged.hu (M.B.); barzo.pal@med.u-szeged.hu (P.B.); 5 HUN-REN-SZTE Neuroscience Research Group, Semmelweis u. 6, H-6725 Szeged, Hungary

**Keywords:** adult, astroglioma, diffuse midline glioma, glioma, histone, H3 K27-altered, prevalence

## Abstract

**Background/Objectives**: Diffuse midline glioma (DMG), H3 K27M-altered, represents a rare group of gliomas arising in midline structures of the central nervous system. Historically regarded as a pediatric entity, it is now increasingly recognized in adults. Although its relative prevalence among all midline diffuse gliomas and its clinical-radiological characteristics are well defined in children, these tumors remain less characterized in adults, and comparative evaluations with H3 K27 wildtype midline diffuse gliomas are limited. **Methods**: Consecutive adult patients with histopathologically confirmed diffuse glioma (WHO grade ≥ 2) diagnosed between 2016 and 2025 were retrospectively screened for midline tumor location, with systematic revision of imaging and pathology. For identified midline diffuse gliomas, comprehensive clinical, imaging, and immunohistochemical data were collected, and a detailed morphometric analysis was performed. H3 K27 alteration status was established immunohistochemically, with supplementary immunostaining when necessary. Descriptive and comparative analyses were conducted. **Results**: A total of 5% of the 541 adult diffuse gliomas were midline, and 23% of IDH wildtype midline gliomas were consistent with DMG, H3 K27-altered (all H3 K27M-mutant). The affected patients were significantly younger, and these tumors predominantly involved the thalamus and mesencephalon. Morphometric analyses revealed trends toward fewer high-grade features in H3 K27-altered tumors, with composite scores demonstrating significant discriminatory ability. The overall survival was not significantly different between groups but showed associations with ring-like enhancement as well as adjuvant and salvage therapies in the overall midline cohort. **Conclusions**: This study provides population-based prevalence estimates for DMG, H3 K27M-altered, and complements the limited literature with comparative clinical-radiological and morphometric data of potential prognostic relevance.

## 1. Introduction

Diffuse midline gliomas (DMGs) represent a rare group of glial tumors that arise in midline structures of the central nervous system (CNS), predominantly involving the thalamus, brainstem, and/or spinal cord. The histopathological features are highly heterogeneous, with the predominant morphology being a diffuse astroglial tumor with high-grade features [1,2]; however, retrospective analyses have also identified low-grade histological phenotypes in a subset of cases [3]. Although biopsy provides essential diagnostic information, it is commonly unfeasible in brainstem cases [4,5], and its routine use in children remains controversial [6]. Similarly, due to the critical anatomical location and infiltrative growth pattern, complete surgical resection is rarely feasible, and even partial resection is often limited [2].

DMGs have a distinct biological behavior that distinguishes them from other types of diffuse gliomas. According to the 2016 and 2021 World Health Organization (WHO) classifications of CNS tumors, DMGs were categorized as pediatric diffuse gliomas (2016) [7] or pediatric-type high-grade diffuse gliomas (2021) [8], reflecting their predominant occurrence in childhood, with a peak incidence at 6–7 years of age [5,9,10]. Historically, these pediatric tumors were referred to as diffuse intrinsic pontine gliomas (DIPGs), reflecting their predominant region of origin. With accumulating data on the molecular background of DIPGs and diffuse pediatric gliomas in other midline locations, the 2016 WHO classification defined these entities as *DMG*, *H3 K27M-mutant*, referring to the characteristic lysine-to-methionine substitution at position 27 in the histone *H3F3A* gene or, less frequently, the related *HIST1H3B* and *HIST1H3C* genes [7]. The recognition of molecular alterations beyond H3 K27M mutations that similarly result in the loss of H3 K27 trimethylation led to the expansion of the term to *DMG*, *H3 K27-altered*, in the 2021 WHO classification [8].

Although DMGs (especially DIPGs) characteristically occur in childhood, recent case reports and series have published H3 K27-altered DMG cases even in the elderly, occasionally in patients >70 years of age [11,12,13,14,15]. In the pediatric population, DMGs account for approximately 10–20% of all brain tumors, 20–40% of all gliomas, and 60–80% of all brainstem gliomas [1,2,16,17]. While the incidence is well documented in children and adolescents, it is less well characterized in adults. In particular, although large cohort studies report that 74–95% of pediatric DMGs (as defined by anatomical location and predominantly comprising DIPGs) are H3 K27-altered [5,18,19,20,21], the data on the relative prevalence of H3 K27 alterations in adult midline diffuse gliomas are limited. Similarly, although the dismal prognosis conferred by H3 K27 alterations in pediatric DMGs is uniformly reported and confirmed by meta-analyses [21], the effect of this molecular background on the survival outcomes in adults are less clear.

With the aim of addressing these gaps regarding the role of H3 K27-altered status in the clinical, radiological, and pathological characteristics of adult midline diffuse gliomas, we conducted a retrospective analysis with supplementary immunohistochemical diagnostic approaches on an exclusively adult cohort.

## 2. Materials and Methods

**Figure 1:**
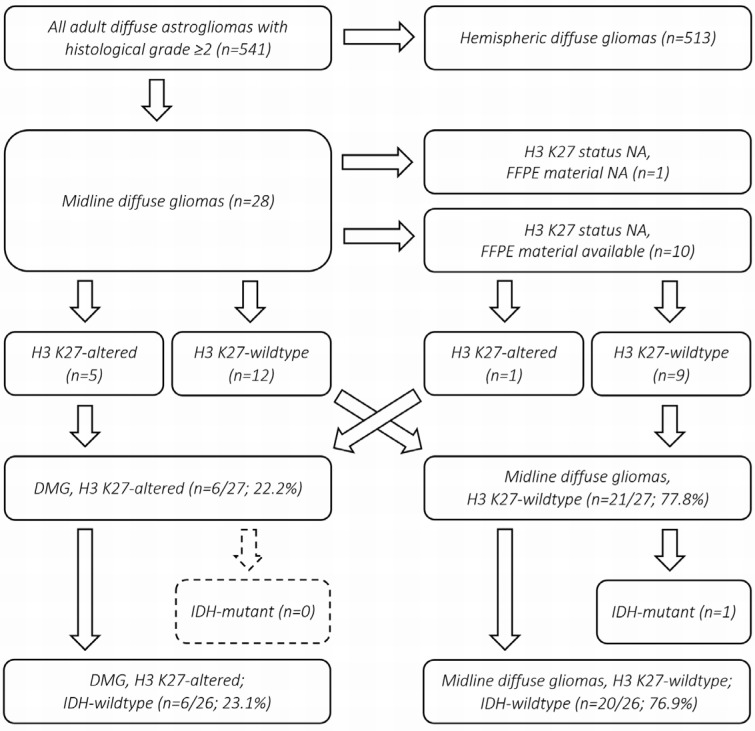
Flowchart illustrating the process of retrospective case identification. The cases with histopathologically confirmed diffuse gliomas (grade ≥ 2) were screened for midline involvement and underwent visual re-evaluation of the initial imaging according to pre-defined criteria. Among midline gliomas, the cases with a previously established H3 K27 alteration status were collected, and additional cases with available FFPE material were included following the supplementary immunohistochemical analysis. The combined set of previously known and newly assessed cases was subsequently filtered based on the IDH status to determine the final sample size for comparative and survival analyses.

We conducted a retrospective identification of DMGs in adult (≥18 years of age) patients in a 10-year period at our tertiary care center. The flowchart of the process is shown in Figure 1. All adult patients with diffuse astroglial tumors (WHO grades 2–4 based on histopathological grounds alone, irrespective of molecular alterations or retrospective CNS WHO assessment) diagnosed histopathologically between January 2016 and November 2025 were systematically screened in the institutional multidisciplinary neuro-oncology tumor board registry. Recurrent tumors for which the first histopathological assessment occurred before the target period were excluded. The cases were filtered to include tumors involving midline anatomical structures, and registry screening was followed by a visual review of preoperative magnetic resonance imaging (MRI) scans to identify tumors located within, centered on, or predominantly involving midline structures. These structures were defined to include the brainstem (medulla oblongata, pons, and mesencephalon), spinal cord, and thalamus, as typical locations listed by CNS WHO, with the inclusion criteria expanded to encompass tumors located in the cerebellum or hypothalamus. Tumors located exclusively in the basal ganglia, pineal region, or corpus callosum were not considered DMGs, similarly to most previously published DMG cohorts [11,12,13,22,23].

For the identified midline diffuse gliomas, the results of immunohistochemical staining for glial fibrillary acidic protein (GFAP), IDH1 R132H, H3K27me3, H3 K27M, alpha-thalassemia/intellectual disability syndrome X-linked (ATRX), tumor protein p53 (p53), and marker of proliferation Kiel 67 (Ki-67) was recorded. In cases lacking the immunohistochemical data on H3 K27 status or in which only H3 K27M immunohistochemistry was available, H3K27me3 immunostaining was retrospectively performed to supplement the diagnosis. The characteristics of the collected immunohistochemical staining, including the supplementary immunostaining, is shown in Table S1. The collected clinical characteristics included age at onset, age at imaging diagnosis, age at surgical histological diagnosis, sex, setting of pathological diagnosis (biopsy or autopsy), presence and degree of surgical resection (gross total or partial, defined by the absence or presence of residual tumor intraoperatively and/or on postoperative MRI, respectively), radiotherapy, chemotherapy, overall survival, and vital status at last follow-up. The collected radiological imaging characteristics included tumor location, the presence and pattern of gadolinium enhancement on T1-weighted sequences (ring-like, diffuse/nodular, mixed, or none), and restricted diffusion on diffusion-weighted imaging (DWI) and apparent diffusion coefficient (ADC) map. A semiquantitative 0–3 score was used to rate the severity of regional involvement on diagnostic MRI, designed to capture the extent of involvement relative to both the tumor size and the size of the affected region. A score of 3 denoted the tumor core, corresponding to the greatest extent of involvement relative to tumor size or a near-total (or total) involvement of a region by an extensive tumor. A score of 2 indicated a substantial involvement not meeting the criteria for score 3. A score of 1 represented minor but observable involvement, whereas a score of 0 indicated no observable involvement. Due to the infiltrative growth pattern of diffuse gliomas and the frequent absence of solid contrast-enhancing component, T2 and fluid-attenuated inversion recovery (FLAIR) sequences were used for this analysis. The ratings and classifications were performed by two neurologists with extensive experience in MRI evaluation, with access to the original neuroradiology reports for reference. The discrepant ratings were resolved by consensus to ensure consistency. Although complete blinding to neuropathology data was not feasible due to the retrospective study design, all evaluations were conducted systematically to minimize potential bias.

For morphometric analysis of available hematoxylin and eosin-stained sections, the following features were evaluated as binary variables (0 = absent, 1 = present): cellular pleomorphism, microvascular proliferation, endothelial hyperplasia, fibrin thrombi, necrosis, pseudopalisading necrosis, visible nucleoli at 10× objective magnification, visible nucleoli at 40× objective magnification, multinucleated tumor cells, microcystic area(s), calcification, vesicular chromatin, and perivascular lymphocytic cuffing. Based on the observed trends in the exploratory analyses and accounting for variable redundancy and missing data, a composite morphometry score (CMS) was retrospectively constructed to summarize morphologic features that showed suggestive associations with the H3 K27 alteration status within this cohort. Features that tended to be inversely associated with H3 K27 alteration, including the presence of *pseudopalisading necrosis and/or endothelial hyperplasia* (combined due to missing data in specimens lacking evaluable vessels), *microvascular proliferation*, and *fibrin thrombi*, were assigned a value of −1, whereas features that tended to be positively associated, namely the presence of *multinucleated tumor cells* and *visible nucleoli at 40× objective magnification*, were assigned a value of +1. The CMS was calculated as the sum of these values, yielding a score ranging from −3 to 2. A discriminatory ability of a simplified CMS, including only *pseudopalisading necrosis*, *microvascular proliferation*, and *fibrin thrombi* was also evaluated. The illustrative scoring sheets outlining the calculation of CMS and simplified CMS are provided in Table S2.

For a statistical analysis, the normality of continuous variables was assessed through Shapiro–Wilk test, and the comparative analyses were done through Student’s *t*-test (applying the Levene’s test and Welch’s correction, where needed) or Mann–Whitney U test, for parameters with normal and non-normal distribution, respectively. Descriptive statistics were reported as mean ± standard error of the mean and median [interquartile range] for normally and non-normally distributed variables, respectively. The effect size (Pearson’s r) was calculated from Cohen’s d by using the r = d/√(d^2^ + 4) formula. Binary categorical variables were compared using Fisher’s exact test, with results expressed as percentages of cases with the feature present, and the effect sizes were reported as Cramer’s V. Ordinal categorical variables were compared using the linear-by-linear association Chi^2^ test with exact *p*-values, with effect sizes reported as Somer’s D. The discriminatory ability of the CMS was evaluated by calculating the area under the receiver operating characteristic (ROC) curve, using the maximized Youden’s J index to obtain the optimal cutoff. The cases with missing data were excluded from the corresponding analyses. The overall survival was defined as the time from the imaging diagnosis to death from any cause, providing an objective measure and allowing for inclusion of autopsy-only cases. Survival according to the H3 K27 status was estimated using the Kaplan–Meier method and compared using the Mantel–Cox log-rank test. Potential prognostic variables were evaluated using univariable Cox proportional hazards models as exploratory analyses. Due to the limited number of events (n < 20), multivariable analyses were restricted to two-variable Cox models to avoid overfitting. All patients were included in the survival analyses, and those who had not experienced the event were treated as censored at their last contact. A *p*-value of <0.05 was considered significant. The SPSS Statistics 22.0 (SPSS Inc., Chicago, IL, USA) software was used.

This retrospective study was reported in accordance with the STROBE statement for observational studies.

## 3. Results

### 3.1. Identification and Classification of Midline Diffuse Gliomas

**Figure 2:**
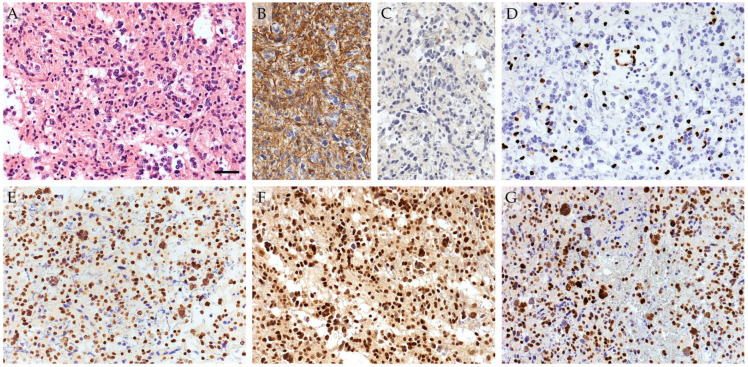
Typical histological and immunohistochemical features of DMG, H3 K27-altered. Hematoxylin and eosin staining demonstrates a diffusely infiltrating glial neoplasm with astrocytic morphology and marked pleomorphism (**A**). GFAP immunohistochemistry confirms glial differentiation (**B**). Immunochemistry for IDH1 R132H canonical mutation is negative (**C**). Tumor cells show loss of H3K27me3 expression (**D**) with strong nuclear positivity for H3 K27M (**E**). ATRX expression is retained (**F**), whereas p53 shows diffuse nuclear positivity, consistent with *TP53* alteration (**G**). Scale bar represents 50 μm.

A total of 541 cases with diffusely infiltrating astroglial tumors were identified, of which 28 (5.2%) met the criteria for a midline tumor (Figure 1). Seventeen had their H3 K27 status documented in the pathology report based on immunohistochemistry (H3K27me3 and/or H3 K27M), five of which were consistent with the diagnosis of DMG, H3 K27-altered (one at autopsy, four by biopsy). Of the remaining 11 cases without documented H3 K27 status, one autopsy case was not available for further work-up. Of the 10 cases with available formalin-fixed, paraffin-embedded (FFPE) material for supplementary immunohistochemistry, one demonstrated H3K27me3 loss, with concordant nuclear immunopositivity for H3 K27M (Figure 2).

These yielded a total of 22.2% (6/27) relative prevalence of DMG, H3 K27-altered, within all midline diffuse gliomas. All H3 K27-altered DMGs and 20/21 H3 K27 wildtype midline diffuse gliomas were negative for IDH1 R132H, yielding a 23.1% (6/26) relative prevalence of DMG, H3 K27-altered, within all IDH wildtype midline diffuse gliomas (in this study defined as negative for canonical IDH1 R132H mutation) in this adult cohort (Figure 1). All H3 K27-altered cases demonstrated immunopositivity for the H3 K27M mutation, whereas all cases negative for H3 K27M immunostaining showed retained H3K27me3 expression. Due to the substantial biological differences between IDH wildtype and IDH-mutant diffuse gliomas (as illustrated by the untreated survival of over 4.5 years in the single IDH1 R132H-mutant midline diffuse glioma case), subsequent analyses were restricted to the IDH wildtype cases.

### 3.2. Demographic and Clinical Characteristics

The comparative clinical and radiological characteristics of the H3 K27-altered and wildtype groups are presented in Table 1. The age at imaging diagnosis was significantly lower in the H3 K27-altered group, with a mean difference of 16.9 years (42.5 ± 8.2 year vs. 59.4 ± 3.3 year, *p* = 0.032). A similar pattern was observed for age at symptom onset, as well as age at surgical histological diagnosis (Table 1). The sex distribution was comparable. The symptom onset was subacute progressive in most cases, with the median time from onset until diagnostic imaging being 1.0 [0.4–2.3] month, without significant between-group difference. However, one H3 K27 wildtype tumor initially presented as a thalamic intracerebral hemorrhage (ICH), substantially delaying the diagnosis (to 10 months). In addition to this ICH, one patient developed lobar ICH during follow-up at the site of a lobar extension.

### 3.3. Radiological Imaging Features

**Figure 3:**
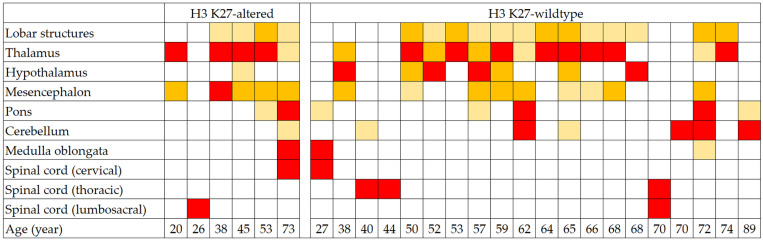
Heatmap illustrating the severity of regional involvement in the CNS of adult patients with midline diffuse gliomas, stratified by the H3 K27 alteration status. The colors denote the degree of involvement, with white representing no involvement (score 0), beige representing mild involvement (score 1), orange representing moderate involvement (score 2), and red representing severe involvement (score 3).

**Figure 4:**
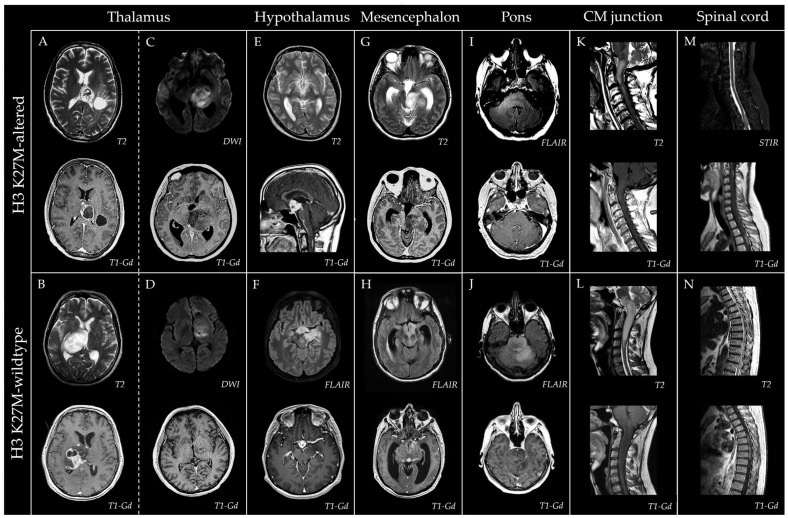
Representative MRI images of adult midline diffuse gliomas. Thalamic gliomas with variable amount and pattern of gadolinium contrast enhancement, including vivid ring-like (**A**), mixed ring-like and solid (**B**), minute nodular (**C**), and minute ring-like enhancement (**D**). Variable extent and intensity of focal restricted diffusion was common (**C**,**D**). A vividly enhancing hypothalamic metastasis of a H3 K27-altered DMG with an initial lumbar spinal presentation, with apparent leptomeningeal enhancement encompassing the brainstem (**E**). Hypothalamic involvement was frequent in H3 K27 wildtype gliomas, here with a contrast-enhancing core and a non-enhancing infiltrative extension to adjacent structures (**F**). Severe mesencephalic involvement in a H3 K27-altered DMG with minimal focal contrast enhancement, leading to hydrocephalus due to cerebral aqueduct compression (**G**). A multifocal H3 K27 wildtype hypothalamic glioma diffusely infiltrating the mesencephalon, similarly leading to hydrocephalus due to cerebral aqueduct obstruction by a solid contrast enhancing component (**H**). Diffusely infiltrating H3 K27-altered DMG involving the pons and extending toward the cerebellum without contrast enhancement (**I**). A strikingly similar pattern in a H3 K27 wildtype case (**J**). Extensive H3 K27-altered DMG with an infiltrative and non-enhancing pattern involving the medulla oblongata and the cervical spinal cord (**K**) and a similar growth pattern in a wildtype glioma (**L**). Multiple diffusely enhancing, solid, leptomeningeal drop metastases in the cervical and thoracic spinal cord of a H3 K27M-altered DMG initially presenting in the lumbar spinal cord (**M**). Focally enhancing H3 K27M wildtype diffuse glioma expanding through the entire thoracolumbar spinal cord, which eventually reached the pons (**N**).

The anatomical distribution and relative severity of regional involvement as observed on diagnostic MRI are summarized in a heatmap shown in Figure 3, with representative images provided in Figure 4.

No significant between-group differences were observed in the frequency of involvement across different CNS regions at this sample size (Table 1). However, a notable trend toward a higher rate of mesencephalic involvement in H3 K27-altered DMGs could be observed (83.3% (5/6) vs. 45.0% (9/20)). This association was supported by the analysis based on the severity of regional involvement, where mesencephalic severity was significantly higher in the H3 K27-altered versus wildtype group (*p* = 0.035), consistent with a greater proportion of severity scores of 2–3 in this region (83.3% (5/6) vs. 30.0% (6/20), Table S3), with score 3 observed exclusively in the H3 K27-altered group (Figure 3). The involvement of the thalamus on diagnostic MRI was nearly universal in the H3 K27-altered group, with the single initially uninvolved case showing subsequent involvement on follow-up. The cerebellar involvement was scarce (16.7%, 1/6) in the H3 K27-altered group, with a mild infiltration observed only in the single most extensive tumor. Contiguous extension into adjacent lobar white matter (often bilateral) was common in both groups. The longitudinal spread of the tumors was usually infiltrative in both groups, frequently without apparent contrast enhancement and occasionally without visible connection to the core. Cases with longitudinally extensive tumors with initial spinal cord involvement were present in both groups. One case in the H3 K27-altered group with an initial tumor located in the conus demonstrated a peculiar upward leptomeningeal spread on follow-up, with multiple contrast-enhancing solid drop metastases throughout the spinal cord and extensive leptomeningeal contrast enhancement surrounding the entire brainstem, eventually giving rise to a solid contrast-enhancing hypothalamic mass with subsequent involvement of bilateral deep hemispheric structures (Figure 4).

On the initial MRI, the frequency and pattern of contrast enhancement and the prevalence of restricted diffusion within tumors did not differ significantly between groups (Table 1). Contrast enhancement was present in 84.6% (22/26) of the total cohort, with ring-like enhancement in 56.0% (14/25) (40.0% (10/25), with and 16.0% (4/25) without additional solid/nodular enhancement). Focal or confluent areas of restricted diffusion were present in 60.9% (14/23) of the total cohort. In line with the hallmark midline location and frequent mesencephalic involvement, hydrocephalus secondary to cerebral aqueduct compression was observed in 3/26 cases (two H3 K27-altered and one wildtype) at presentation (11.5%). Representative radiological images of adult midline diffuse gliomas are shown in Figure 4.

### 3.4. Histopathological Diagnosis and Treatment

A total of 2/26 patients (7.7%; one per group) had autopsy only, and had not previously received anti-tumor treatment. Ten out of 26 patients (38.5%) had biopsy and 14/26 (53.8%) had tumor resection, without between-group differences. The majority of the resections were partial (12/14, 85.7%), whereas two patients (both wildtype) had gross total resection. In addition to the two autopsy patients, one resection and two biopsy patients did not receive oncologic therapy due to frailty, and three were lost to follow-up (treated abroad). Radiotherapy according to the Stupp regimen was initiated in 78.3% (18/23), with concomitant temozolomide therapy in 69.6% (16/23). Adjuvant temozolomide and salvage bevacizumab therapies were initiated in 47.8% (11/23) and 34.8% (8/23), respectively. Although no significant between-group differences were observed in treatment variables, the H3 K27-altered group tended to receive adjuvant and salvage therapies more frequently (Table 1).

### 3.5. Histopathological Features

Figure 2 illustrates a typical histopathological and immunohistochemical profile of a DMG, H3 K27-altered. Two H3 K27 wildtype specimens were not available for morphometric analyses, and in one H3 K27-altered case, endothelial hyperplasia could not be assessed due to overt necrosis. While the histopathological features of H3 K27-altered gliomas were rather heterogeneous, the vast majority of H3 K27 wildtype diffuse midline gliomas corresponded to the histopathological diagnosis of glioblastoma multiforme (GBM), WHO grade 4. In the exploratory univariable comparative morphometric analyses, endothelial hyperplasia was found to be significantly associated with H3 K27 wildtype tumors (present in 88.9% (16/18) vs. 40.0% (2/5) in the H3 K27-altered group), without further significant associations at this sample size. However, notable trends suggested microvascular proliferation, fibrin thrombi, necrosis, and pseudopalisading necrosis to be somewhat more frequent in the H3 K27 wildtype group, whereas the presence of visible nucleoli at 40× objective magnification and multinucleated tumor cells were nominally more common in H3 K27-altered gliomas (Table 2). Constructed retrospectively from exploratory morphometric variables coded as positive or negative according to their observed trends with H3 K27 alteration status, the CMS was significantly associated with H3 K27-altered tumors within this cohort (*p* = 0.012, Somer’s D = 0.329). The ROC analysis supported the discriminatory ability of CMS, yielding an area under the curve (AUC) of 0.838 (95% confidence interval (CI) 0.629–1.000; *p* = 0.015, Figure S1). At a suggested optimal cutoff of −0.5, the CMS values ≥ 0 were indicative of H3 K27-altered tumors, with a sensitivity of 83.3% and specificity of 88.9%. A simplified CMS using only microvascular proliferation, pseudopalisading necrosis, and fibrin thrombi assigned a value of −1 produced slightly less but acceptable discriminatory potential (AUC = 0.782 (95% CI 0.550–1.000), *p* = 0.042, Figure S1), with an optimal cutoff of −1.50, corresponding to a sensitivity of 83.3% and a specificity of 72.2% for identifying H3 K27 alteration at values of ≥−1.

### 3.6. Overall Survival

At the time of the analysis, four out of six patients (66.7%) in the H3 K27-altered group and 15 of 20 patients (75.0%) in the wildtype group had died. The median overall survival from diagnostic imaging was 21.9 m (95% CI 20.5–23.3) in the H3 K27-altered versus 9.7 m (95% CI 6.2–13.2) in the wildtype group, which did not yield statistical significance through the log-rank test (Table 1). The Kaplan–Meier curve is shown in Figure S2. Given the lack of observable differences between altered and wildtype groups, the exploratory analyses were performed by fitting univariable Cox regression models on clinical and radiological variables to assess their potential effect on survival in the total cohort (Table 3). These identified ring-like contrast enhancement (increased hazard), adjuvant temozolomide therapy (decreased hazard), and salvage bevacizumab therapy (decreased hazard) as factors having significant association with overall survival, with notable trends for age (trend toward increased hazard), cervical spinal cord involvement (trend toward increased hazard), and concomitant temozolomide therapy (trend toward decreased hazard, Table 3). Hazard ratio and *p*-value for autopsy-only histopathology could not be estimated by Cox regression because the variable produced near-complete separation, reflecting the rapidly progressive disease course in autopsy cases, in which no diagnosis or treatment could be established during life (see corresponding Kaplan–Meier curve in Figure S3). After adjusting for age at diagnostic imaging in separate multivariable Cox proportional hazards models, salvage bevacizumab therapy remained the only variable significantly associated with survival (Table 3). Autopsy-only histopathology produced a similar near-complete separation in the age-adjusted Cox model.

## 4. Discussion

Diffuse midline glioma was first defined as a distinct entity in the 2016 WHO Classification of Tumors of the CNS among pediatric diffuse gliomas, characterized by the presence of an H3 K27M missense mutation in histone H3.1 or H3.3 variant genes (*HIST1H3B/C* or *H3F3A*), resulting in a lysine-to-methionine substitution at position 27 of the histone H3 protein [7]. The H3.3 variant is more frequently affected, accounting for approximately 70% of H3 K27M-mutant cases, whereas H3.1 mutation is observed in about 30% [1]. Over the past decade, studies have demonstrated that H3 K27 alterations represent an early and initiating event in the oncogenesis of DMGs, a relationship most clearly established in DIPG. Under physiological conditions, the polycomb repressive complex 2 (PRC2) catalyzes the methylation of histone H3 at lysine 27 (H3 K27) via its catalytic subunit, enhancer of zeste homolog 2 (EZH2). Trimethylation of H3 K27 maintains chromatin compaction and represses gene expression. Disruption of this methylation signal due to loss of trimethylation leads to global transcriptional dysregulation, facilitating activation and amplification of multiple proto-oncogenes.

The first identified pathophysiological background of the loss of H3 K27 trimethylation was the H3 K27M substitution, which prevents trimethylation at lysine 27, resulting in widespread epigenetic dysregulation that promotes oncogenesis [1]. Even a small fraction of H3 K27-mutant histones (3–17%) is sufficient to induce widespread chromatin effect through a self-reinforcing mechanism, whereby the mutated K27 residue suppresses PRC2 activity itself by occupying the catalytic subunit, EZH2 [1,24]. The resulting global hypomethylation of H3 K27, in turn, drives widespread transcriptional alterations. In addition to the canonical H3 K27M mutation, rare cases harboring an H3 K27I substitution have been reported [19,25].

Subsequent studies have demonstrated that the loss of H3 K27 trimethylation may also occur through alternative molecular mechanisms, such as overexpression of the enhancer of zeste homologs inhibitory protein (EZHIP), which inhibits PRC2 by also targeting its catalytic subunit, EZH2. Consequently, the entity was renamed DMG, H3 K27-altered in the 5th edition of the WHO Classification of Tumors of the CNS [8]. The overexpression of the EZHIP as a cause of the loss of H3 K27 trimethylation has been observed in approximately 15% of DMGs [1]. This frequently co-occurs with mutations or amplifications of the (epidermal growth factor receptor) *EGFR*, which are predominantly found in bithalamic pediatric DMGs [26,27].

In addition to H3 K27 alterations, a high incidence of concurrent tumor protein 53 (*TP53*) mutations and alterations in growth factor signaling pathways, including activin A receptor type I (*ACVR1*) and platelet-derived growth factor receptor alpha (*PDGFRA*), has been reported in many pediatric DMG cases [28]. Additional alterations may involve alpha-thalassemia/intellectual disability syndrome X-linked (*ATRX*), components of the mitogen-activated protein kinase (MAPK) signaling pathway, neuroblastoma-derived MYCN (N-MYC), and the cyclin-dependent kinase inhibitor 2A/2B (*CDKN2A/B*) tumor suppressor genes. B-Raf proto-oncogene serine/threonine-protein kinase (*BRAF*) V600E mutation and IDH mutations are exceptional [1,22,29,30,31].

Based on accumulating experimental evidence, the predilection of H3 K27-altered DMGs for midline CNS structures most likely reflects a cell-of-origin-dependent tumorigenic effect, in which certain glial progenitor populations (including oligodendrocyte precursor-like cells) exhibit selective vulnerability to H3 K27 alteration due to spatiotemporally determined (i.e., anatomical location- and age-specific) cell-intrinsic and cell-extrinsic (microenvironmental) factors rather than reflecting any anatomical specificity of the mutation itself [3,32].

Although H3 K27-altered DMGs predominantly occur in children, reflecting an age-associated tumorigenic effect, there is increasing recognition that this tumor type can also arise in adults, including the elderly. While the relative prevalence of DMG, H3 K27-altered, among all midline diffuse gliomas, as well as their clinical and radiological characteristics, are well-documented in pediatric populations, the data in adults are limited. The current literature is largely restricted to single case reports [10,14,15,33]; studies on mixed pediatric–adult cohorts [23,34]; studies on DMG series limited to specific anatomical location(s) [34,35,36,37,38,39]; studies on isolated adult H3 K27-altered DMG series without a wildtype comparator group [4,12,28,40], with a comparator group comprising or including hemispheric gliomas [13,41], with a comparator group including significant amount of grade I and/or non-astroglial midline tumors [11], with a selected (i.e., not specified as population-based) wildtype comparator group [13], or reporting very small cohorts with limited comparisons [41]. We are aware of only three prior studies that provide presented or calculable data on the relative prevalence of H3 K27-altered DMGs among all midline diffuse gliomas in consecutive, population-based cohorts [22,42,43] using somewhat variable anatomical and grade-related inclusion criteria (one of which included a negligible number of IDH-mutant or grade I cases in the comparator [42]). Although comparative survival data are reported in these studies, between-group characterization remains limited. In order to provide population-based epidemiological data and to address the gaps in the comparative clinical, radiological, and histopathological characterization of adult midline diffuse gliomas, we conducted a retrospective, single-center analysis of an unselected, consecutive cohort of adult diffuse glioma cases, comparing features between H3 K27-altered and wildtype tumors.

In our consecutive cohort, 5.2% of all diffuse gliomas met our criteria for midline diffuse glioma. This rate is lower than the 19.2% reported in a previous study including midline cortex, corpus callosum, and a few grade I tumors in addition to regions and grades present in our criteria [42] but aligns more with other studies using inclusion criteria similar to ours, which reported rates of 3.7–4.4% [41,43]. We found a 22.2% relative prevalence of DMG, H3 K27-altered, within all adult midline diffuse gliomas in our cohort, which increased to 23.1% when excluding the IDH-mutant case from the H3 K27 wildtype group. This prevalence falls within the range of 14.6–47.8% reported in the three prior studies, which used slightly variable definitions, yielding a weighted average of 23.6% across the four studies, based on aggregate case counts. These values are consistently lower than those observed in pediatric populations, yet remain remarkable in light of the historical perception that these tumors occur almost exclusively in children. In our cohort, all cases that demonstrated the loss of H3K27me3 harbored the H3 K27M mutation by immunohistochemistry. Moreover, all cases with only a negative H3 K27M immunostaining available in the archives showed retained H3K27me3 on supplementary immunohistochemistry, excluding additional H3 K27-altered cases related to less frequent mechanisms, such as the EZHIP overexpression or H3 K27I mutation, which were not directly addressed. This approach was novel, as prior epidemiology-focused studies did not incorporate H3K27me3 status into their case definition [22,41,42,43].

With respect to demographic characteristics, previous studies have shown consistent trends indicating that H3 K27-altered DMGs present at a younger age compared to wildtype midline diffuse gliomas, with the largest sample-size cohort demonstrating statistical significance [22,41,42,43]. Additionally, most of these studies describe a consistent, though individually non-significant, trend for a slight male preponderance in DMG, H3 K27-altered, as also highlighted in a recently published literature review [44]. These findings are consistent with our data, with a significantly lower age and a trend toward male predominance in DMG, H3 K27-altered, compared to wildtype midline gliomas.

As previously reported, H3 K27-altered DMGs predominantly involve the brainstem or thalamic region, with a trend toward thalamic predominance [4,12,22,23,40,42,43,45]. Similarly, in our study, the tumors most frequently arose in these locations. Additionally, the severity of mesencephalic involvement was significantly greater in H3 K27-altered DMGs vs. wildtype midline diffuse gliomas, a finding and an approach that, to our knowledge, have not been previously described in the literature.

On post-gadolinium T1-weighted MRI sequences, contrast enhancement was reported in more than 50% of cases by most authors, frequently in a ring-like pattern [1,6,13,42,46]. This trend was also observed in our cohort, albeit the spectrum of enhancement ranged from zero to vivid and encompassed patchy nodular, diffuse solid, ring-like, and mixed patterns in variable combinations. Importantly, the extent of a contrast-enhancing lesion on MRI often did not delineate the tumors’ boundaries, as it many times became evident on follow-up MRIs as well, especially since a non-enhancing, infiltrative, T2 hyperintense growth pattern was rather common, and the enhancement often affected only a small proportion of the lesion. These need to be accounted for when assessing the extent of surgical resection in midline gliomas. Leptomeningeal spread is occasionally reported [12,13,33,40,47,48] and could also be observed in one of our patients with an initial conus tumor. Systematic data on the presence of restricted diffusion on MRI in adult DMG are lacking in the literature. We observed a high rate of focal or diffuse restricted diffusion, albeit of mild-to-moderate intensity, in midline gliomas without significant between group difference. In a study of 31 pediatric patients, no difference was observed between H3 K27-mutant and wildtype tumors in diffusion characteristics, which is consistent with our findings [18].

Histopathological systematic morphometric data are sparsely reported in the literature; however, a general observation is that H3 K27-mutant DMGs do not uniformly exhibit high-grade features. Our observation on the 50% prevalence of necrosis and 40% prevalence of endothelial hyperplasia resonates well with 41% and 52% rates observed by others [11]. In fact, endothelial hyperplasia was significantly less frequent in the H3 K27-mutant group. Analyzed together with other high-grade morphological features such as microvascular proliferation, pseudopalisading necrosis, and fibrin thrombi, alterations that are otherwise typical in classical GBMs, the retrospectively created composite scores effectively discriminated the H3 K27-altered group from the wildtype midline gliomas. Given the retrospective construction and the relatively small sample size, the results of these composite scores should clearly be regarded as exploratory and need confirmation by future studies but align well with prior observations regarding the possibility of lower grade morphological appearances in DMG, H3 K27-altered.

In our cohort, H3 K27-altered DMGs were not associated with worse prognosis, with a mean overall survival of 22.7 months (nominally even better than in the wildtype comparator), which is comparable to or slightly exceeding that reported in previous studies [13,22,23,28,39,40,41]. In adults, a number of studies have found that the loss of H3 K27 trimethylation is not consistently associated with poorer outcomes [11,13,22,41,42,43], particularly in supratentorial tumors, and one even reported a slightly better prognosis in the H3 K27-altered group [42]. However, it should be noted that the survival characteristics can be biased by the relatively younger age of patients with H3 K27-altered DMGs, as observed across several studies. In our exploratory analysis of potential predictors of survival, the significant association with autopsy diagnosis likely reflects an aggressive disease course that precluded the initiation of invasive diagnostic procedures and treatment in our cohort. The consistent trends for adjuvant temozolomide and especially salvage bevacizumab therapies (the later retaining significance in the age-adjusted analysis as well) resonates with prior observations of a study on brainstem H3 K27M-altered DMGs regarding adjuvant therapies [39]. However, caution should be taken when interpreting these results as potential causative associations, as at least in our cohort, patients with a less progressive course and longer clinical stability were more likely to receive further treatments, which might as well indicate an inverse causative relationship behind these associations.

Our study has several limitations, including the retrospective design, the relatively small sample size despite the 10-year study period, the restriction of supplementary analyses to cases with available FFPE material, incomplete patient follow-up in a subset of cases, and reliance on immunohistochemical approaches without complementary molecular analyses, all of which render some of our observations exploratory and hypothesis-generating in nature. The limited sample size likely reduced the statistical power to detect differences in overall survival. The reliance on IDH1 R132H immunohistochemistry may have overlooked rare non-canonical IDH1/IDH2 mutations, which could affect prevalence estimates within tumors classified as ‘IDH wildtype’. The strengths of the study include systematic data collection from an unselected cohort of consecutive patients, enabling the calculation of population-based estimates, including relative prevalence of DMG, H3 K27M-altered, among all midline diffuse gliomas, as well as robust comparative analyses across a comprehensive set of clinical, radiological, and histopathological parameters.

## 5. Conclusions

Our retrospective, population-based, comparative analyses revealed a 23.1% relative prevalence of H3 K27-altered DMGs among IDH wildtype adult midline diffuse gliomas; supported their thalamic–mesencephalic predominance by demonstrating near-universal involvement at diagnosis; provided evidence for high rates but variable patterns and intensity of contrast enhancement and restricted diffusion on MRI; identified a lower frequency and potential discriminatory value of high-grade microvascular changes on histopathology; and found no statistically significant impact of H3 K27-altered status on the overall survival in the adult population.

## Figures and Tables

**Table 1:** Demographic and clinicoradiological characteristics of adult midline diffuse gliomas.

	H3 K27-Altered	H3 K27 Wildtype	Subject No.	*p*-Value	ES
*Demographic characteristics*					
**Age at symptom onset (y)**	42.3 ± 8.2	59.2 ± 3.5	6 vs. 19	**0.038**	0.428
**Age at diagnostic imaging (y)**	42.5 ± 8.2	59.4 ± 3.3	6 vs. 20	**0.032**	0.434
**Age at surgical histological diagnosis (y)**	36.3 ± 6.6	59.0 ± 3.4	5 vs. 19	**0.006**	0.608
Sex (male/all %)	66.7 (4)	55.0 (11)	6 vs. 20	1.000	0.099
*Regions involved on MRI*					
Lobar structures (%)	66.7 (4)	65.0 (13)	6 vs. 20	1.000	0.015
Thalamus (%)	83.3 (5)	65.0 (13)	6 vs. 20	0.628	0.167
Hypothalamus (%)	16.7 (1)	35.0 (7)	6 vs. 20	0.628	0.167
Brainstem (total, %)	83.3 (5)	55.0 (11)	6 vs. 20	0.352	0.245
Mesencephalon (%)	83.3 (5)	45.0 (9)	6 vs. 20	0.170	0.324
Pons (%)	33.3 (2)	25.0 (5)	6 vs. 20	1.000	0.079
Medulla oblongata (%)	16.7 (1)	10.0 (2)	6 vs. 20	1.000	0.088
Cerebellum (%)	16.7 (1)	30.0 (6)	6 vs. 20	1.000	0.127
Spinal cord (total, %)	33.3 (2)	20.0 (4)	6 vs. 20	0.596	0.133
Spinal cord (cervical, %)	16.7 (1)	5.0 (1)	6 vs. 20	0.415	0.184
Spinal cord (thoracic, %)	0.0 (0)	15.0 (3)	6 vs. 20	1.000	0.198
Spinal cord (lumbosacral, %)	16.7 (1)	5.0 (1)	6 vs. 20	0.415	0.184
*Qualitative MRI findings*					
Hydrocephalus (%)	33.3 (2)	5.0 (1)	6 vs. 20	0.123	0.374
Contrast enhancement (%)	66.7 (4)	90.0 (18)	6 vs. 20	0.218	0.272
Ring-like enhancement * (%)	40.0 (2)	60.0 (12)	5 vs. 20	0.623	0.161
Solid/nodular enhancement * (%)	40.0 (2)	75.0 (15)	5 vs. 20	0.283	0.300
Area(s) with restricted diffusion (%)	75.0 (3)	57.9 (11)	4 vs. 19	1.000	0.133
*Tissue sampling and treatment*					
Autopsy (%)	16.7 (1)	5.0 (1)	6 vs. 20	0.415	0.184
Biopsy (%)	16.7 (1)	45.0 (9)	6 vs. 20	0.352	0.245
Resection (%)	66.7 (4)	50.0 (10)	6 vs. 20	0.652	0.141
Gross total resection (%)	0.0 (0)	10.0 (2)	6 vs. 20	1.000	0.158
Radiotherapy (%)	83.3 (5)	76.5 (13)	6 vs. 17	1.000	0.073
Concomitant temozolomide (%)	83.3 (5)	64.7 (11)	6 vs. 17	0.621	0.178
Adjuvant temozolomide (%)	83.3 (5)	35.3 (6)	6 vs. 17	0.069	0.422
Salvage bevacizumab (%)	66.7 (4)	23.5 (4)	6 vs. 17	0.131	0.398
*Survival*					
OS from imaging (m)	21.9	9.7	6 vs. 20	0.482	0.667

* Including mixed patterns. For binary variables, the data are presented as percentages (absolute numbers) of cases with the feature present, with *p*-values from Fisher’s exact test and effect sizes as Cramer’s V. For age at diagnostic imaging, the data are presented as mean ± SEM, with *p*-values from Student’s *t*-test and effect sizes as Pearson’s r. For survival analysis, the data are presented as median survival (in months), with *p*-value from the Mantel–Cox log-rank test and effect size as point estimates of the hazard ratio from Cox proportional hazards regression. Statistical significance is indicated in bold. Italics denote variable categories. ES, effect size.

**Table 2:** Histopathological morphometric characteristics of adult midline diffuse gliomas.

	H3 K27-Altered	H3 K27 Wildtype	Subject No.	*p*-Value	ES
Cellular pleomorphism (%)	83.3 (5)	94.4 (17)	6 vs. 18	0.446	0.174
Microvascular proliferation (%) *^,#^	50.0 (3)	88.9 (16)	6 vs. 18	0.078	0.415
Endothelial hyperplasia (%) *	40.0 (2)	88.9 (16)	5 vs. 18	**0.048**	**0.489**
Fibrin thrombi (%) *^,#^	16.7 (1)	61.1 (11)	6 vs. 18	0.155	0.385
Necrosis (%)	50.0 (3)	88.9 (16)	6 vs. 18	0.078	0.415
Pseudopalisading necrosis (%) *^,#^	16.7 (1)	61.1 (11)	6 vs. 18	0.155	0.385
Microcystic area(s) (%)	16.7 (1)	5.6 (1)	6 vs. 18	0.446	0.174
Visible nucleoli at 10× magnification (%)	16.7 (1)	5.6 (1)	6 vs. 18	0.446	0.174
Visible nucleoli at 40× magnification (%) *	66.7 (4)	44.4 (8)	6 vs. 18	0.640	0.192
Multinucleated tumor cells (%) *	83.3 (5)	50.0 (9)	6 vs. 18	0.341	0.293
Calcification (%)	16.7 (1)	11.1 (2)	6 vs. 18	1.000	0.073
Vesicular chromatin (%)	66.7 (4)	77.8 (14)	6 vs. 18	0.618	0.111
Perivascular lymphocytic cuffing (%)	33.3 (2)	22.2 (4)	6 vs. 18	0.618	0.111
**CMS (score ≥ 0, %)**	83.3 (5)	11.1 (2)	6 vs. 18	**0.012**	**0.329**
**Simplified CMS (score ≥ −1, %)**	83.3 (5)	27.8 (5)	6 vs. 18	**0.031**	**0.298**

* Variables contributing to CMS. ^#^ Variables contributing to simplified CMS. Data are presented as percentages (absolute numbers) of cases with the feature present, with *p*-values from Fisher’s exact test and effect sizes as Cramer’s V. For illustrative purposes, CMS data are presented as percentage (absolute number) of cases with a score above cutoff, while *p*-values and effect sizes were calculated from the raw score (linear-by-linear association Chi^2^, Somer’s D). Statistical significance is indicated in bold.

**Table 3:** Cox proportional hazards models for overall survival from imaging diagnosis in adult midline diffuse gliomas.

	Univariable	Univariable	Age-Adjusted	Age-Adjusted
	*p*-Value	HR (95% CI)	*p*-Value	HR (95% CI)
*Demographic characteristics*				
Age at diagnostic imaging	0.065	1.026 (0.998–1.054)	-	-
Sex	0.348	0.618 (0.227–1.687)	0.053	0.308 (0.093–1.016)
H3 K27 status	0.485	0.667 (0.214–2.079)	0.643	1.399 (0.339–5.771)
*Regions involved on diagnostic MRI*				
Lobar structures	0.589	1.301 (0.501–3.382)	0.978	1.014 (0.386–2.666)
Thalamus	0.709	0.829 (0.309–2.220)	0.746	0.848 (0.311–2.306)
Hypothalamus	0.488	1.417 (0.529–3.796)	0.227	1.957 (0.658–5.818)
Brainstem (total)	0.943	0.967 (0.380–2.462)	0.731	0.845 (0.325–2.201)
Mesencephalon	0.862	0.921 (0.364–2.331)	0.996	1.002 (0.392–2.565)
Pons	0.676	0.767 (0.221–2.666)	0.050	0.205 (0.042–1.003)
Medulla oblongata	0.531	0.522 (0.068–3.998)	0.179	0.229 (0.027–1.967)
Cerebellum	0.381	1.560 (0.577–4.218)	0.640	0.730 (0.196–2.723)
Spinal cord (total)	0.871	0.912 (0.298–2.788)	0.690	1.269 (0.393–4.104)
Spinal cord (cervical)	0.055	15.540 (0.942–256.393)	0.067	13.375 (0.831–215.380)
Spinal cord (thoracic)	0.625	0.692 (0.158–3.030)	0.714	0.758 (0.172–3.345)
Spinal cord (lumbosacral)	0.956	0.959 (0.218–4.221)	0.739	1.293 (0.286–5.847)
*Qualitative MRI findings*				
Hydrocephalus	0.269	0.433 (0.098–1.907)	0.516	0.594 (0.123–2.862)
Contrast enhancement	0.894	1.089 (0.312–3.793)	0.807	0.852 (0.235–3.085)
Ring-like enhancement *	**0.022**	**3.987 (1.221–13.021)**	0.062	3.251 (0.942–11.218)
Solid/nodular enhancement *	0.549	0.736 (0.271–2.003)	0.659	0.795 (0.286–2.208)
Area(s) with restricted diffusion	0.497	0.707 (0.260–1.923)	0.766	0.854 (0.303–2.408)
*Tissue sampling and treatment*				
**Autopsy**	**4 × 10^−8^**	**NCS**	**NCS**	**NCS**
Biopsy	0.803	1.135 (0.419–3.078)	0.841	0.901 (0.325–2.499)
Resection	0.228	0.558 (0.216–1.443)	0.463	0.693 (0.261–1.844)
Gross total resection	0.228	2.551 (0.556–11.706)	0.887	1.141 (0.185–7.036)
Radiotherapy	0.229	0.499 (0.161–1.549)	0.794	0.842 (0.231–3.061)
Concomitant temozolomide	0.086	0.418 (0.154–1.131)	0.684	0.734 (0.166–3.250)
**Adjuvant temozolomide**	**0.013**	**0.286 (0.107–0.767)**	0.086	0.292 (0.072–1.189)
**Salvage bevacizumab**	**0.005**	**0.204 (0.068–0.614)**	**0.020**	**0.163 (0.035–0.750)**

* Including mixed patterns. CI, confidence interval; HR, hazard ratio; NCS, near-complete separation where *p*-value and HR could not be estimated by Cox regression, whereas univariable *p*-value was obtained from Mantel–Cox log-rank test for this variable. Statistical significance is indicated in bold. Italics denote variable categories.

## Data Availability

The original contributions presented in this study are included in the article/Supplementary Material. Further inquiries can be directed to the corresponding author.

## Supplementary Materials

The following supporting information can be downloaded at: https://www.mdpi.com/article/10.3390/pathophysiology33010021/s1; Table S1: Specification of the primary antibodies used for immunohistochemistry (IHC); Table S2: Illustrative scoring sheets demonstrating the calculation of CMS and simplified CMS; Table S3: Regional severity scores on diagnostic MRI of adult midline diffuse gliomas according to H3 K27 alteration status; Figure S1: ROC curves illustrating the discriminatory ability of CMS and simplified CMS for predicting H3 K27 alteration status in adult midline diffuse gliomas; Figure S2: Kaplan–Meier survival curves illustrating overall survival from diagnostic imaging stratified by H3 K27 alteration status in adult midline diffuse gliomas; Figure S3: Kaplan–Meier survival curves illustrating the overall survival from diagnostic imaging stratified by ring-like enhancement, autopsy status, adjuvant temozolomide therapy, and salvage bevacizumab therapy in adult midline diffuse gliomas.

## Abbreviations

The following abbreviations are used in this manuscript:

ACVR1	activin A receptor type I
ADC	apparent diffusion coefficient
AUC	area under the curve
ATRX	alpha-thalassemia/intellectual disability syndrome X-linked
BRAF	B-Raf proto-oncogene serine/threonine-protein kinase
CDKN2A/B	cyclin-dependent kinase inhibitor 2A/2B
CI	confidence interval
CM	cervicomedullary junction
CMS	composite morphometry score
CNS	central nervous system
DIPG	diffuse intrinsic pontine glioma
DMG	diffuse midline glioma
DWI	diffusion-weighted imaging
EGFR	epidermal growth factor receptor
EZHIP	enhancer of zeste homologs inhibitory protein
EZH2	enhancer of zeste homolog 2
FFPE	formalin-fixed, paraffin-embedded
FLAIR	fluid-attenuated inversion recovery
GBM	glioblastoma multiforme
H3 K27M	lysine-to-methionine substitution at position 27 of histone H3
H3K27me3	trimethylation of histone H3 at lysine 27
Ki-67	marker of proliferation Kiel 67
MAPK	mitogen-activated protein kinase
MRI	magnetic resonance imaging
N-MYC	neuroblastoma-derived MYCN
PDGFRA	platelet-derived growth factor receptor alpha
PRC2	polycomb repressive complex 2
p53	tumor protein p53
R132H	arginine-to-histidine substitution at amino acid position 132
ROC	receiver operating characteristic
STIR	short tau inversion recovery
*TP53*	tumor protein 53 gene
T1-Gd	gadolinium-enhanced T1
WHO	World Health Organization
