# Supplementary material for: Prevalence and Clinicoradiopathological Characterization of H3 K27-Altered Diffuse Midline Gliomas in Adults—A Retrospective Observational Study
Source: Pathophysiology. 2026 Mar 14;33(1):21. doi: 10.3390/pathophysiology33010021 (PMC13028753; doi:10.3390/pathophysiology33010021)
Supplement: Supplementary file 1 [file pathophysiology-33-00021-s001.zip › pathophysiology-4170627-supplementary.pdf]

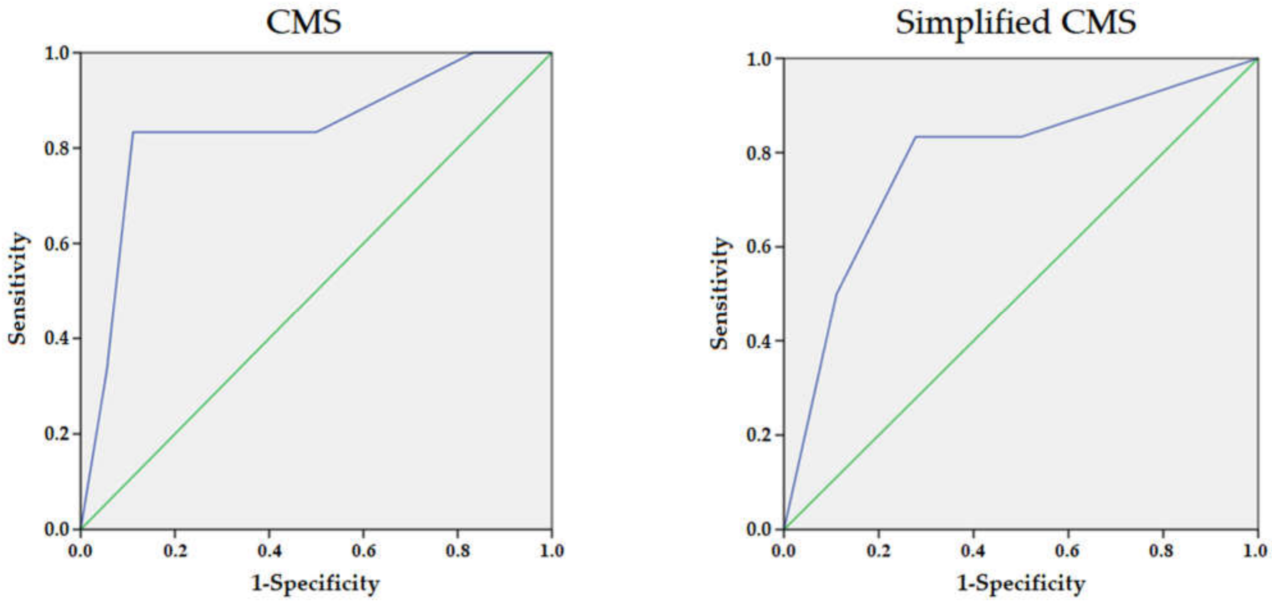

Figure S1. ROC curves illustrating the discriminatory ability of CMS and simplified CMS for predicting H3 K27 alteration status in adult midline diffuse gliomas. CMS, composite morphometry score; ROC, receiver operating characteristic.

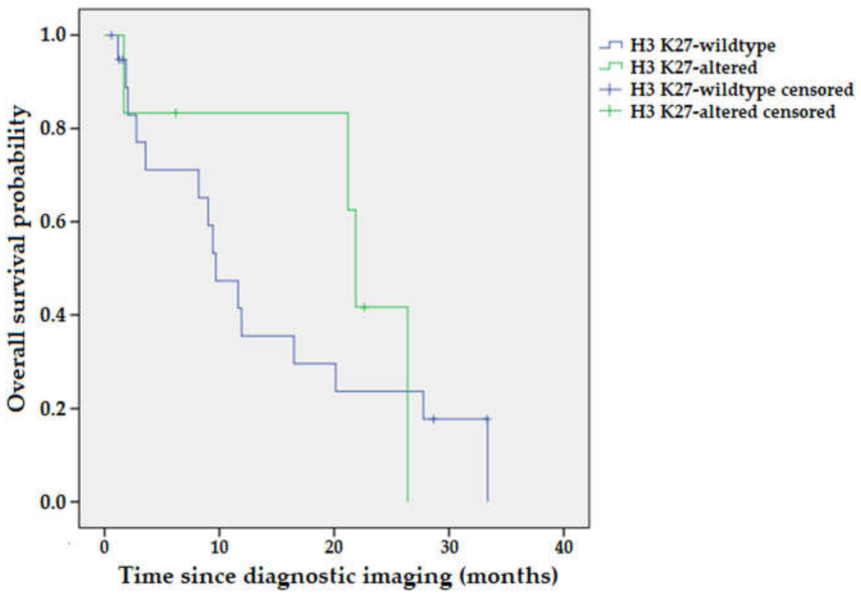

Figure S2. Kaplan-Meier survival curves illustrating overall survival from diagnostic imaging stratified by H3 K27 alteration status in adult midline diffuse gliomas.

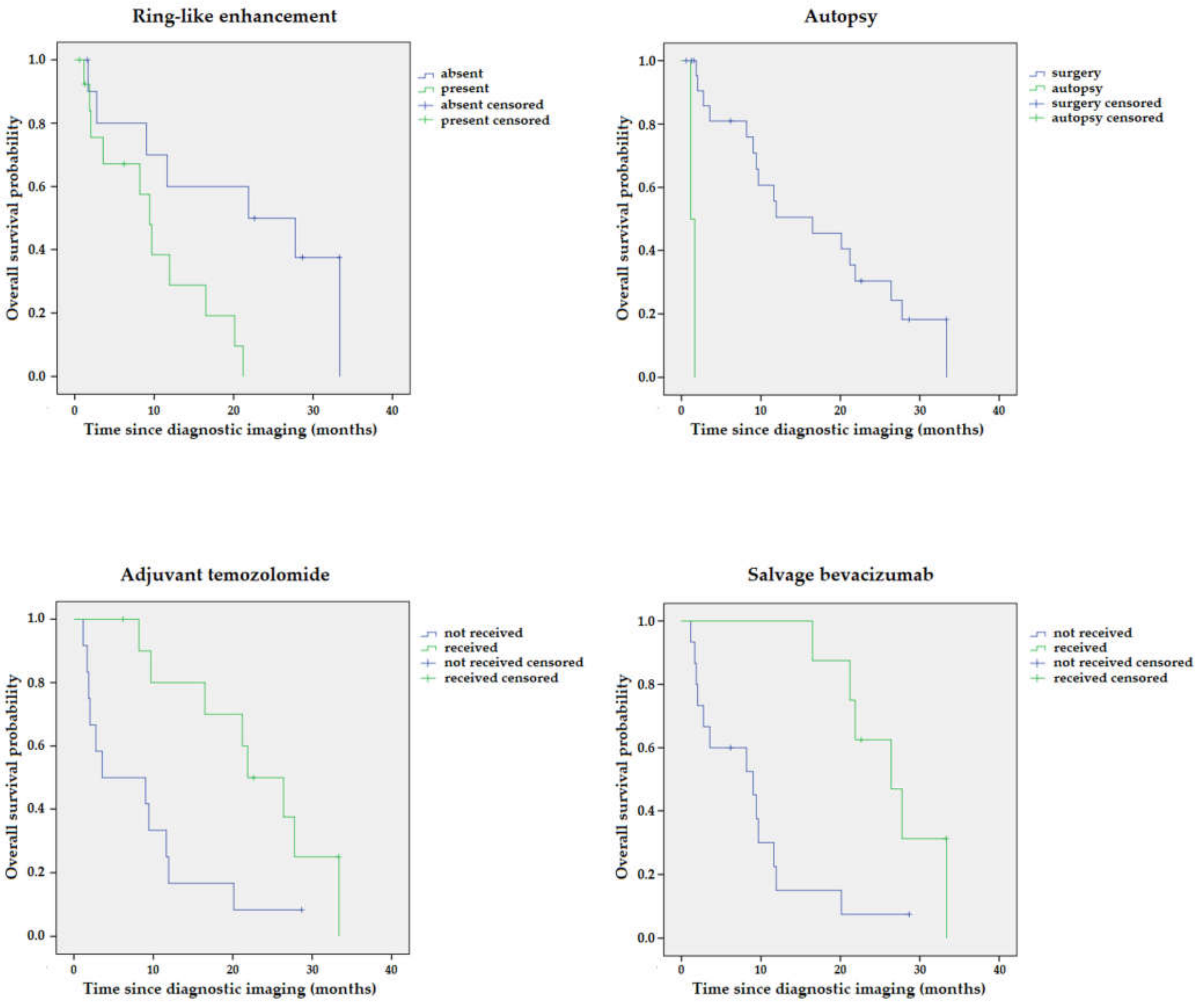

Figure S3. Kaplan-Meier survival curves illustrating the overall survival from diagnostic imaging stratified by ring-like enhancement, autopsy status, adjuvant temozolomide therapy, and salvage bevacizumab therapy in adult midline diffuse gliomas.

Table S1. Specification of the primary antibodies used for immunohistochemistry (IHC). The reactions were performed on Leica Bond III automated IHC staining system.

Antibody	Clone	Manufacturer	Dilution
GFAP	G-A-5	BioSB	1:2000
IDH1 R132H	H09	Dianova	1:400
ATRX	polyclonal rabbit	Sigma-Aldrich (Merck)	1:300
p53	DO7	DAKO	1:1 ready-to-use
Ki-67	MIB-1	DAKO	1:1 ready-to-use
H3K27me3	EP233	BioSB	1:1 ready-to-use
H3 K27M	polyclonal rabbit	Millipore	1:800

ATRX, alpha-thalassemia/mental retardation syndrome X-linked protein; GFAP, glial fibrillary acidic protein; H3 K27M, lysine-to-methionine at position 27 of histone H3 protein; H3K27me3, trimethylation at lysine 27 of histone H3; IDH1 R132H, arginine-to-histidine substitution at position 132 of isocitrate dehydrogenase 1; Ki-67, marker of proliferation Kiel 67; p53, tumor protein p53.

Table S2. Illustrative scoring sheets demonstrating the calculation of CMS and simplified CMS.

Morphometric feature	Present = 1, Absent = 0
Microvascular proliferation	–
Endothelial hyperplasia and/or pseudopalisading necrosis	–
Fibrin thrombi	–
Visible nucleoli at 40x objective magnification	+
Multinucleated tumor cells	+
**CMS (sum)**	∑

Morphometric feature	Present = 1, Absent = 0
Microvascular proliferation	–
Pseudopalisading necrosis	–
Fibrin thrombi	–
**Simplified CMS (sum)**	∑

CMS values ≥ 0 were indicative of H3 K27-altered tumors, with a sensitivity of 83.3% and specificity of 88.9% in our cohort. Simplified CMS values ≥ –1 (i.e. practically no more than one feature present) were indicative of H3 K27-altered tumors, with a sensitivity of 83.3% and specificity of 72.2% in our cohort. CMS, composite morphometry score.

Table S3. Regional severity scores on diagnostic MRI of adult midline diffuse gliomas according to H3 K27 alteration status.

	H3 K27-altered	H3 K27-wildtype	Subject no.	p-value	ES
Lobar structures	0.83 ± 0.31	0.95 ± 0.18	6 vs 20	0.781	-0.040
Thalamus	2.17 ± 0.54	1.60 ± 0.30	6 vs 20	0.394	0.138
Hypothalamus	0.17 ± 0.17	0.90 ± 0.29	6 vs 20	0.246	-0.178
Brainstem (total)	2.00 ± 0.45	1.05 ± 0.26	6 vs 20	0.116	0.218
Mesencephalon	1.83 ± 0.40	0.75 ± 0.20	6 vs 20	**0.035**	**0.318**
Pons	0.67 ± 0.49	0.45 ± 0.21	6 vs 20	0.832	0.076
Medulla oblongata	0.50 ± 0.50	0.20 ± 0.16	6 vs 20	0.854	0.127
Cerebellum	0.17 ± 0.17	0.70 ± 0.27	6 vs 20	0.491	-0.138
Spinal cord (total)	1.00 ± 0.63	0.60 ± 0.28	6 vs 20	0.596	0.133
Spinal cord (cervical)	0.50 ± 0.50	0.15 ± 0.15	6 vs 20	0.415	0.292
Spinal cord (thoracic)	0.00 ± 0.00	0.45 ± 0.25	6 vs 20	0.562	-0.261
Spinal cord (lumbosacral)	0.50 ± 0.50	0.15 ± 0.15	6 vs 20	0.415	0.292

Data are presented as mean ± SEM, with p-values obtained from the exact linear-by-linear association Chi^2 test and effect sizes reported as Somer's D. H3 K27, lysine 27 residue of histone H3; MRI, magnetic resonance imaging, SEM, standard error of the mean.
